# Supplementary material for: The interplay between foraging choices and population growth dynamics
Source: PLoS One. 2025 Jun 26;20(6):e0325942. doi: 10.1371/journal.pone.0325942 (PMC12200681; doi:10.1371/journal.pone.0325942)
Supplement: S1 File — Sensitivity of the probability of finding food and the utility function. (PDF) [file pone.0325942.s001.pdf]

Supplementary Material
The interplay between foraging choices and population growth dynamic

Jimmy Calvo-Monge[1], Baltazar Espinoza*[2], Fabio Sanchez[3] Jorge Arroyo-Esquivel[4]

1 Centro de Investigación en Matemática Pura y Aplicada, Universidad de Costa Rica, Ciudad Universitaria Rodrigo Facio, San José, 11501, Costa Rica

2 Biocomplexity Institute, University of Virginia, Virginia, USA

3 Escuela de Matemática-CIMPA, Universidad de Costa Rica, Ciudad Universitaria Rodrigo Facio, San José, 11501, Costa Rica

4 University of California Davis, California, USA

A The probability of finding food function

In this section, we explore the impact of changes in the probability of changing food. We assume that the probability of finding food is expected to increase as the foraging time increases, where the population density determines the marginal benefits of increasing foraging. Food competition at high population densities is assumed to produce low marginal benefits at low foraging levels, requiring high foraging times to significantly increase the probability of finding food. In contrast, low population densities are assumed to yield high marginal benefits at low foraging times, resulting in a high probability of finding food with shorter foraging times. This mathematical component adds great flexibility to our model. Figure A1 illustrates the assumed family of probability of finding food functions $P^{FF}(f, P(t))$.

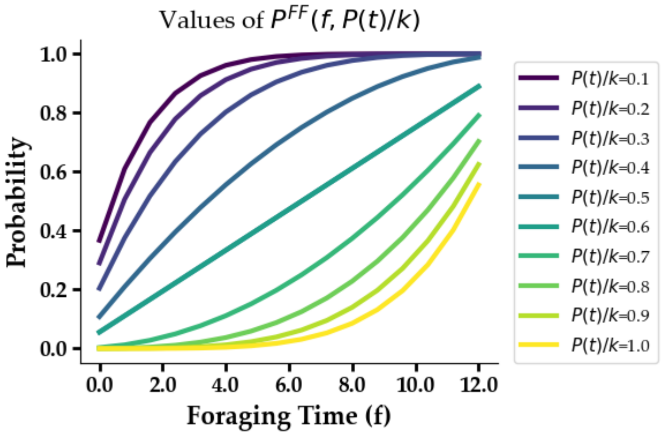

Figure A.1: **Probability of finding food P^{FF} as a function of foraging time selection.** We use the proportion of population size per carrying capacity $P(t)/K$ as the parameter to normalize P^{FF} The probability of finding food increases with longer foraging times. However, the marginal benefits decrease as the population grows. We use the Beta cumulative density function to derive the family of curves.

These functional families can be obtained through several mathematical artifacts. In this study, we use the cumulative distribution functions of Beta distributions computed as

$$F(x;\alpha,\beta) = \frac{B(x;\alpha,\beta)}{B(1;\alpha,\beta)}, \quad \text{where} \quad B(x;\alpha,\beta) = \int_0^x t^{\alpha-1}(1-t)^{\beta-1}dt,$$

is the incomplete Beta function [1]. The α and β parameters control the curve's steepness. Therefore, we can

use the value of $P(t)$ to modify them. The function is then translated to have domain $\mathcal{F} = [f_{\min}, f_{\max}]$. Another approach to obtain the $P^{FF}(f, P(t))$ functions can be the use of sigmoid functions.

B The utility function

The utility shape is also a mathematical feature of the adaptive approach and can be modified with considerable versatility. The standard quadratic function presented in the main article and first used in [2] can be replaced with alternative functional forms that exhibit different marginal benefits. For example, a sigmoid function of the shape

$$s(f) = \alpha/(1 + e^{\theta(f_{\text{opt}} - f)}) \tag{B.1}$$

depicted in Fig.B.2. For this function, higher θ values yield a more sensitive population, unlike the case of the quadratic formulation, where higher ν parameters are interpreted as less sensitive populations.

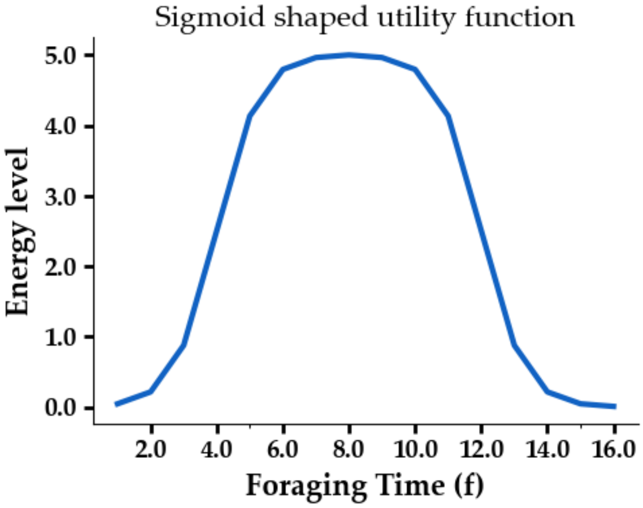

Figure B.2: **Sigmoid-shaped utility function** Utility function with sensitivity parameter $\theta = 1.55$ obtained as a reflection of two sigmoid functions across the $f = f_{\text{opt}}$ axis. In this functional form, the marginal benefits are minimal at both low and high foraging times. Intermediate foraging times produce the maximal marginal benefits.

We conducted simulations similar to those in the main article using a sigmoid-shaped utility function, and our results show that adaptation is less evident or pronounced with this functional form. Fig. B.3 shows a sensitive population forced to keep relatively high foraging times and suboptimal energy levels. In contrast, Fig. B.4 illustrates that a population with a lower sensitivity can sustain optimal energy levels engaging in lower foraging times. This outcome is similar to the situation discussed in our main results.

Although the formulation of the adaptive behavior follows the standard procedures of Markov Decision Processes, this example shows that the model is sensitive to variations in individual-level tradeoffs determined by the functional forms of the probability of finding food and the utility function. This flexibility allows the model to capture a broader range of ecological scenarios and population attributes; however, it also increases the complexity and cost of accurate modeling.

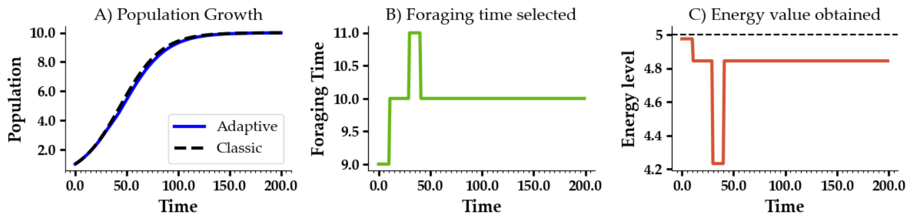

Figure B.3: **Population dynamics high foraging time.** Population growth, optimal daily foraging time selected, and net daily energy gain for an adaptive logistic growth process the utility function attains a maximum value of $u = 5$ at $f_{\text{opt}} = 8$ and uses a sigmoid function with a sensitivity parameter of $\theta = 1.7$. Parameters $\tau = 7, \delta = 0.98, b = 16, k = 10$ and $r_c = 0.01$.

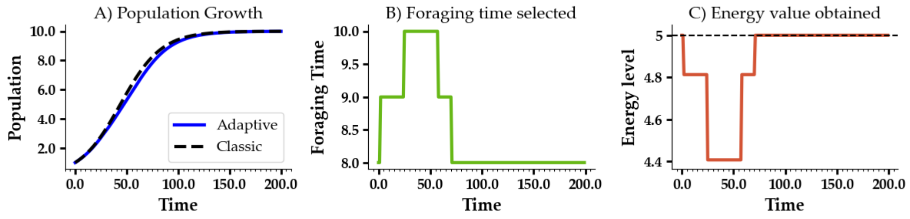

Figure B.4: **Population dynamics low foraging time.** Population growth, optimal daily foraging time selected, and net daily energy gain for an adaptive logistic growth process with parameters $\tau = 7, \delta = 0.98, b = 16, k = 10$ and $r_c = 0.01$. The utility function attains a maximum value of $u = 5$ at $f_{\text{opt}} = 8$ and uses a sigmoid function with a sensitivity parameter of $\theta = 0.9$.

Finally, for highly sensitive agents (low values of ν), the population growth trajectory for the adaptive approach does not provide a substantial difference compared to the classical approach. In these cases, the utility function is almost flat, producing low marginal benefits of increasing the foraging time. It follows that there is no significant energy gain across foraging time options. Conversely, the difference between the classic and adaptive logistic models becomes null for a low-sensitive population (high values of ν). The agents do not perceive enough incentives to adapt their behavior in these scenarios. In summary, the adaptive framework characterizes individual-level behavioral trade-offs for which the aggregated decisions can influence population growth.

C Sensitivity of the probability of finding food

In this section, we explore the impact of variations in the probability of finding food on the foraging time choices. The probability of finding food for each foraging time selection depends on the changing status of the population dynamics. S1 Fig 1 offers a heuristic approach to define such probabilities a priori. The definition of these probability functions is a very important factor that influences the outcome of the adaptive process. For example, if we increase the general probability values to have a minimum value of $P = 0.8$, as in S3 Fig 1.

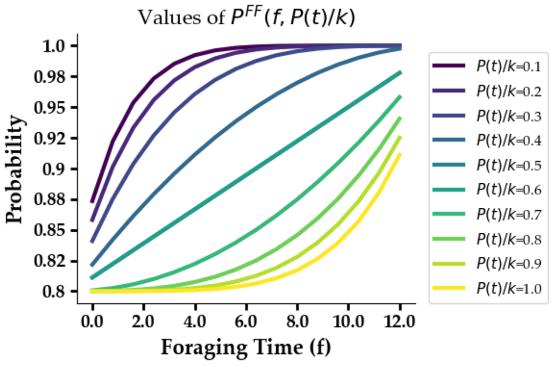

Figure C.5: **Modified probability of finding food** Values of the function P^{FF} for each foraging time selection, as the population size $P(t)$ increases, using a the same approach as in Figure A.1, however adding a minimum value of $P = 0.8$.

A process using the exact parameters of S2 Fig 1 of the main article but using this new P^{FF} function gives a very different result, as seen in S3 Fig 2, where the energy strain placed on the foraging agents is less severe (precisely because of the increase in the probability of finding food for each foraging selection). The circumstances described in S3 Fig 1 reflect a population with more advantageous conditions than one with probabilities given in S1 Fig 1. These variations could result from environmental or communal attributes, which make it more/less probable to find food at a given foraging time.

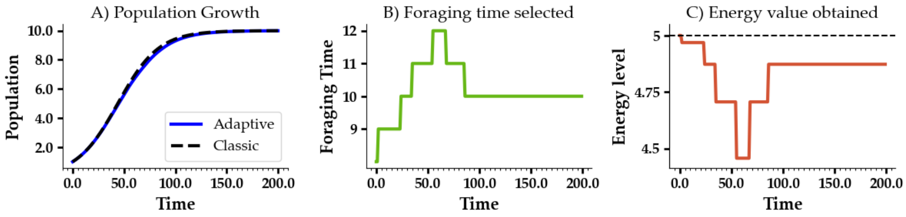

Figure C.6: **Population dynamics with modified probability of finding food.** Population growth, optimal daily foraging time selected and net daily energy gain for an adaptive logistic growth process with parameters $\nu = 0.4, \tau = 7, \delta = 0.98, b = 16, k = 10$ and $r_c = 0.01$. In this case, we use the probability of finding food $P^{FF}(f, P(t))$ given by Fig C.5, which has higher overall probabilities. Compare this to the corresponding simulation in the main article, which uses the same parameters. Higher probabilities of finding food incur less energy demands from decision agents.

References

[1] Morris H. DeGroot and Mark J. Schervish. *Probability and Statistics.* Addison-Wesley, Boston, 4th edition, 2012.

[2] Eli P. Fenichel, Carlos Castillo-Chavez, M. Graziano Ceddia, Gerardo Chowell, Paula A. Gonzalez Parra, Graham J. Hickling, Garth Holloway, Richard Horan, Benjamin Morin, Charles Perrings, et al. Adaptive human behavior in epidemiological models. *Proceedings of the National Academy of Sciences*, 108(15):6306–6311, 2011.
