# Supplementary material for: The interplay between foraging choices and population growth dynamics
Source: PLoS One. 2025 Jun 26;20(6):e0325942. doi: 10.1371/journal.pone.0325942 (PMC12200681; doi:10.1371/journal.pone.0325942)
Supplement: Attachment — Submitted filename: reviewer_response_letter.pdf [file pone.0325942.s002.pdf]

May 5, 2025

Charlotte Chibuzor Ndiribe, Ph.D.
Academic Editor
PLOS ONE

Dear Editor,

We thank the handling editor and the reviewers for the time and effort invested in evaluating our manuscript ID **PONE-D-25-06482**. In what follows, we have addressed each reviewer's comments point-by-point in italics and describe our response in blue. Changes have been included in the main manuscript using blue text. Additionally, we request that Dr. Baltazar Espinoza serve as the corresponding author (baltazar.espinoza@virginia.edu) for the manuscript. We have also included as coauthor Dr. Jorge Arroyo-Esquivel, an expert in mathematical ecology who helped address one of the main concerns of the reviewers regarding the ecological research context.

We hope that the current draft has adequately responded to the helpful comments of the reviewers and it is acceptable for publication in PLOS ONE.

Response to the Editor's comments

This is a timely work with a new model for combining foraging and theoretical logistic growth models to elucidate the dynamics of populations, particularly individual foraging decisions and the impacts on growth. This would be of broad encompassing utility across vast biological systems.However, what clearly stands out is the lack of in-depth ecological analyses demonstrating applicable or transferable knowledge across some key populations existing within families of the taxonomic kingdoms. In alignment with the reviewers, more ecological research on foraging behaviour should rightly be featured in this study for the wider benefit of ecologists. Also, I strongly recommend the manuscript is edited for English quality, beginning from the abstract.

Response: We have included examples of empirical and modeling work on behavioral ecology, particularly in foraging theory and how they support the results of this paper in the Introduction and Discussion sections to put this work further in the context of foraging theory. Specifically, we added the following paragraph in the introduction section.

The connection between individual decision-making and ecological dynamics lies at the core of behavioral ecology [1, 2, 3, 4, 5]. Various modeling frameworks have been proposed to address the central challenge of understanding how collective dynamics emerge from individual decisions. Life history theories [6, 7, 8, 9], theory of reproductive strategies [10, 11], social foraging theory [12, 13, 14, 15, 16], and Optimal Foraging Theory (OFT) [17, 18, 19] constitute a robust theoretical foundation for studying individual foraging decisions. However, these frameworks are often not well suited to describing fine-scale, short-term behavioral traits that depend on environmental conditions, such as predation and starvation risks. In their pioneering work, Clark and Mangel introduced a modeling framework that approaches behavior from an evolutionary perspective within the context of an individual's life history, using a dynamic state-variable approach. This framework has been successfully applied to a wide range of ecological questions, including group size in social carnivores, host selection, clutch size in parasitic insects, seasonal survival strategies, and avian migration, among other paradigms [19, 20, 21]. In this study, we build on this foundational framework and couple it with the logistic model to study the trade-off between individual-level reproductive and behavioral traits and their influence on population growth.

and the following paragraph in the discussion section

These results are consistent with empirical observations of migratory species that rely on resources that provide low energy per individual unit. In such cases, like that of leatherback turtles, the additional energy ex-

pended migrating to areas of high prey density is offset by the benefits, even if it results in reduced reproductive growth [22]. This trade-off does not appear in species with sufficiently high fat reserves to sustain their reproductive efforts [23]. Other examples of adaptive behaviors that involve balancing individual traits include: the activity patterns of zebrafish (Danio rerio) which exhibit lower movement rates in dark environments and higher rates in illuminated ones [24]; insects decisions when selecting feeding or oviposition sites [21]; and the group hunting strategies and habitat use observed in African lions (Panther leo) [25, 26].

Abstract: '... the logistic growth model to account for dynamic incentives depending on [delete the] population size. Our results yield insights into the multiscale processes, such as __ and ___, that impact population robustness beyond mere size and aggregated reproductive traits.' This variation may be induced by environments imposing distinct behavioral cost-benefit trade-offs that require individuals to exert distinct levels of [efforts on what?]

We edited the abstract to include the following lines:

- Our results provide insights into multiscale processes–such as selection pressure of behavioral choices and the cost-benefit of social activities–that influence population robustness beyond mere size and aggregated reproductive traits.
- This variation may be induced by environments imposing distinct behavioral cost-benefit trade-offs that require individuals to exert different levels of foraging effort to maintain reproductive viability.

Introduction: Line 17: Remove "has" emphasised. Line 19: Remove "have" utilised. Line 21: Similar tense issue. Line 24: "lie in". Line 35: With a bracket explain agent, few words. Line 49: "insights into" or "on information transfer across scales". Others: Line 52, 60, 68 with "envision", 78 "time effort"?, 79 "individual-level processes", 82 delete "the", many others with "the" issues. Line 97, this is not a proposal, Line 102-110 rewrite to meet contemporary standards.

Thank you for the helpful comment. We have done an editorial pass of the entire manuscript, and these comments have been addressed in the current version.

Methods: Write early sentences and first paragraph well. Clearly distinguish Sampling strategy from the statistical development. Line 136: "We may use" Rephrase, please.

In this framework there is no sampling strategy or statistical development. The decision-making process we use is a dynamic optimization problem, as described in the main manuscript: "The daily decision-making process poses a dynamic optimization problem centered on determining the optimal allocation of time and energy for searching for resources necessary for survival"

Results: The first sentence begs for clarity, what are you referring to? Remove unnecessary words/ statements here and throughout this work. Rewrite results section with proper captions, other than figures. Figures only depict a set of tests, those are your captions. Again, it is important to adhere to modern styles of writing science. Rightly, tables and figures are bracketed.

This has been addressed in the current version of the manuscript.

Discussions: You need test organisms or about 2-3 study systems as references to support and validate the utility of your model as claimed. Again, check and correct all grammatical lapses here.

Thank you for the comment. This has been addressed and highlighted in blue in the main manuscript. In particular, as noted in our response to a previous comment, we added the paragraph beginning with the line: *These results are consistent with empirical observations...*

Conclusions: This should not start again with a repeat "In conclusion".

This has been addressed in the current version of the manuscript. Additionally, we have extended the conclusion sections.

Response to the Reviewer's comments

Reviewer #1: Fabio Sanchez and his collaborators explore theoretically the interplay between individual-level behavior and population growth dynamics. Although the text and analyses look solid and convincing, I realized that my (limited) background in theoretical ecology does not allow me to thoroughly evaluate the paper and, in particular, the models presented. However, one problem I noticed is that the authors do not discuss much how their modeling framework could be used in real biological systems. I would appreciate if some examples were given (I think this modeling framework could be used for many different systems, including plant or insect populations). It could also be emphasized how the models should be parameterized for different systems.

Thank you for the helpful comment. This has been addressed in the main manuscript and the details have been discussed in previous points.

Reviewer #2: Overview Sanchez et al. have developed a model that integrates a foraging model into the logistic growth model. This is an interesting model that provides some novel insights into the impact of the 'average' individual-level response on population growth. I believe the model developed is most certainly worthy of publication, but I feel the authors have overlooked a wealth of ecological research on foraging behaviour that would greatly benefit, provide insights to the practitioner of how this model could be used, and the ecological literature could perhaps inform the model parameterization.

Thank you for the helpful comment. This has been addressed in the main manuscript and the details have been discussed in previous points.

General Comments

The model develop is very interesting and I think adds to the general ecological modelling literature. However, as noted above, there is a wealth of literature related to foraging behaviour that is not discussed in any level of detail here and I think would benefit the paper. Putting this model in the context of what has been already done will enhance the relevance and impact of this paper on the ecological literature. For example, the shape of the foraging function and the probability of finding food which underlie the model could be based on different species life-histories, such as ambush predators, generalist predators, grazing predators, scavengers, etc. By doing this, the impact of this method on the population growth rates of different 'types' of life-histories could be captured. In classical ecological literature, the idea of a functional response is well established and should be discussed whenever talking about modelling foraging behaviour (e.g. Holing, https://esajournals.onlinelibrary.wiley.com/doi/pdf/10.1890/0012-9623-95.3.200). Also, in the marine realm ideas around foraging arena theory likely has relevance to this work. (see a number of Carl Walters papers or https://pressbooks.bccampus.ca/ewemodel/chapter/foraging-arena-theory/).

We have included some empirical and modeling examples of foraging theory and how the results of this paper are supported by them in the Introduction and Discussion sections to put this work further in the context of foraging theory. The details have been discussed in previous points.

Specific Comments

Line 168: It is not clear to me what the 'planning horizon' represents (also give it the symbol τ). As a result, on line 234, what planning effort means, in ecological terms, is unclear.

The concept of "planning horizon" in this work is analogous to the classic concept used in optimization theory. It refers to the length of time into the future considered when making decisions. Specifically, the dynamic optimization process used to model behavioral choices weights current and expected future benefits over the planning period $[t, t+\tau]$. To obtain the optimal foraging time at time t, we use the *Bellman's principle of optimality* and split the dynamic optimization problem over the planning horizon into a sequence of subproblems over sequential periods $[t, t+1]$, $[t+1, t+2]$,$\cdots$,$[t+\tau-1, t+\tau]$. We added the following lines in the "Materials and methods" section: *The chosen behavioral decision balances potential immediate foraging cost and energy gains, as well as discounted expected cost-benefit tradeoffs over the planning horizon-the period during which a representative individual evaluates the cost-benefit trade-offs that guide its behavioral choices.*

Additionally, we didn't find the term "planning effort" in our main manuscript. Perhaps the reviewer meant "foraging effort"? If so, foraging effort refers to the time individuals invest searching for food.

Lines 184-192: I think it would be useful to show the utility function underlying the models for Figure 2 and 3 in the main text. I would suggest showing these two utility functions in Figure 1.

We edited Figs. 2 and 3 to show the the associated utility functions driving the distinct scenarios.

Line 186: replace "stale' with "stable".

This has been corrected in the main manuscript.

Lines 200-218 are more of a discussion that results.

We appreciate this comment. We have moved some of these lines to the discussion section.

Line 200-201: Simplify this to "Figs 2 and 3 show how this framework visualizes processes. . . ."

This has been addressed in the main manuscript.

Line 205: "Resilience" in ecological terms generally is related to the ability of a population to recover from low abundance, or to sustain itself in the face of some detrimental change in the environment, so I would avoid that word here. It is more that the impact of this 'life history' on the population growth is less that the impact of the life history in Figure 2.

We edited the main manuscript as follows: *This indicates that populations with lower behavioral sensitivity (i.e., less reactive to environmental or social cues) are less affected by environmental changes because their life-history traits reduce their dependency on immediate conditions. As a result, they are more adaptable to density-induced resource limitations.*

Line 213: Rather than 'energy drain', I would suggest 'foraging effort'.

This has been edited in the text.

For the supplemental section, it would be nice in the methods to link to the relevant sections of the methods.

We edited the main manuscript accordingly.

Sincerely,

Fabio Sanchez, Ph.D.

References

[1] Dora Biro, Takao Sasaki, and Steven J Portugal. Bringing a time–depth perspective to collective animal behaviour. *Trends in ecology & evolution*, 31(7):550–562, 2016.

[2] Deborah M Gordon. The evolution of the algorithms for collective behavior. *Cell systems*, 3(6):514–520, 2016.

[3] Andrew J King, Gaëlle Fehlmann, Dora Biro, Ashley J Ward, and Ines Fürtbauer. Re-wilding collective behaviour: an ecological perspective. *Trends in ecology & evolution*, 33(5):347–357, 2018.

[4] Peter AH Westley, Andrew M Berdahl, Colin J Torney, and Dora Biro. Collective movement in ecology: from emerging technologies to conservation and management, 2018.

[5] Iain D Couzin, Jens Krause, et al. Self-organization and collective behavior in vertebrates. *Advances in the Study of Behavior*, 32(1):10–1016, 2003.

[6] George F Oster and Edward O Wilson. *Caste and ecology in the social insects*. Princeton University Press, 1978.

[7] John Maynard-Smith. *Models in ecology*. Cambridge university press, 1974.

[8] Alan Grafen. Natural selection, kin selection and group selection. *Behavioural ecology: An evolutionary approach*, 1986.

[9] Jon E Brommer. The evolution of fitness in life-history theory. *Biological Reviews*, 75(3):377–404, 2000.

[10] Eric R Pianka. On r-and k-selection. *The american naturalist*, 104(940):592–597, 1970.

[11] Mart R Gross. Alternative reproductive strategies and tactics: diversity within sexes. *Trends in Ecology & Evolution*, 11(2):92–98, 1996.

[12] Thomas Caraco and Luc-Alain Giraldea. Social foraging: producing and scrounging in a stochastic environment. *Journal of Theoretical Biology*, 153(4):559–583, 1991.

[13] Ariana Strandburg-Peshkin, Danai Papageorgiou, Margaret C Crofoot, and Damien R Farine. Inferring influence and leadership in moving animal groups. *Philosophical Transactions of the Royal Society B: Biological Sciences*, 373(1746):20170006, 2018.

[14] Larissa Conradt and Timothy J Roper. Group decision-making in animals. *Nature*, 421(6919):155–158, 2003.

[15] Odile Petit and Richard Bon. Decision-making processes: the case of collective movements. *Behavioural processes*, 84(3):635–647, 2010.

[16] Luc-Alain Giraldeau and Frédérique Dubois. Social foraging and the study of exploitative behavior. *Advances in the Study of Behavior*, 38:59–104, 2008.

[17] Graham H Pyke, H Ronald Pulliam, and Eric L Charnov. Optimal foraging: a selective review of theory and tests. *The quarterly review of biology*, 52(2):137–154, 1977.

[18] David W Stephens and John R Krebs. *Foraging theory*, volume 6. Princeton university press, 1986.

[19] Marc Mangel and Colin W Clark. Towards a unifield foraging theory. *Ecology*, 67(5):1127–1138, 1986.

[20] Marc Mangel and Colin Whitcomb Clark. *Dynamic modeling in behavioral ecology*, volume 8. Princeton University Press, 1988.

[21] Colin W Clark and Marc Mangel. *Dynamic state variable models in ecology: methods and applications.* Oxford University Press, 2000.

[22] Bryan P. Wallace, Michael Zolkewitz, and Michael C. James. Discrete, high-latitude foraging areas are important to energy budgets and population dynamics of migratory leatherback turtles. *Scientific Reports*, 8(1), July 2018.

[23] Jerome Casas, Gerard Driessen, Nicole Mandon, Sebastiaan Wielaard, Emmanuel Desouhant, Jacques Van Alphen, Laurent Lapchin, Ana Rivero, Jean Philippe Christides, and Carlos Bernstein. Energy dynamics in a parasitoid foraging in the wild. *Journal of Animal Ecology*, 72(4):691–697, July 2003.

[24] RC MacPhail, J Brooks, DL Hunter, B Padnos, TD Irons, and S Padilla. Locomotion in larval zebrafish: Influence of time of day, lighting and ethanol. *Neurotoxicology*, 30(1):52–58, 2009.

[25] Justin P Suraci, Laurence G Frank, Alayne Oriol-Cotterill, Steve Ekwanga, Terrie M Williams, and Christopher C Wilmers. Behavior-specific habitat selection by african lions may promote their persistence in a human-dominated landscape. *Ecology*, 100(4):e02644, 2019.

[26] Scott R Loarie, Craig J Tambling, and Gregory P Asner. Lion hunting behaviour and vegetation structure in an african savanna. *Animal Behaviour*, 85(5):899–906, 2013.
